# Longitudinal Monitoring of the Effects of Anti-Adenoviral Treatment Regimens in a Permissive In Vivo Model

**DOI:** 10.3390/v16081200

**Published:** 2024-07-26

**Authors:** Ann E. Tollefson, Anna Cline-Smith, Jacqueline F. Spencer, Baoling Ying, Dawn M. Reyna, Elke Lipka, Scott H. James, Karoly Toth

**Affiliations:** 1 Department of Molecular Microbiology and Immunology, Saint Louis University School of Medicine, St. Louis, MO 63104, USA; ann.tollefson@health.slu.edu (A.E.T.); anna.smith@health.slu.edu (A.C.-S.); jacqueline.spencer@health.slu.edu (J.F.S.); bying@wustl.edu (B.Y.); 2 TSRL, Inc., Ann Arbor, MI 48108, USA; dreyna@tsrlinc.com (D.M.R.); elipka@tsrlinc.com (E.L.); 3 Division of Pediatric Infectious Diseases, Department of Pediatrics, University of Alabama at Birmingham, Birmingham, AL 35233, USA; sjames@uabmc.edu

**Keywords:** adenovirus, antiviral, animal model, hamster, luciferase

## Abstract

Adenovirus infections of immunocompromised patients can cause life-threatening disseminated disease. While there are presently no drugs specifically approved to treat these infections, there are several compounds that showed efficacy against adenovirus in preclinical studies. For any such compound, low toxicity is an essential requirement. As cumulative drug effects can accentuate pathology, especially in patients with other morbidities, it is important to limit antiviral exposure to what is absolutely necessary. This is achievable by monitoring the virus burden of the patients and administering antivirals to suppress virus replication to a non-pathogenic level. We modeled such a system using Syrian hamsters infected with a replication-competent adenovirus vector, in which luciferase expression is coupled to virus replication. We found that virus replication could be followed in vivo in the same animal by repeated measurement of luciferase expression. To test the utility of an interrupted treatment regimen, we used NPP-669 and valganciclovir, two antiviral compounds with high and moderate anti-adenoviral efficacy, respectively. We found that short-term treatment of adenovirus-infected hamsters at times of peak virus replication can prevent virus-associated pathology. Thus, we believe that this animal model can be used to model different treatment regimens for anti-adenoviral compounds.

## 1. Introduction

Adenovirus (HAdV) infections are ubiquitous among humans. The seven species and more than one hundred types of the virus cause a diverse array of disease [1,2,3]. The illnesses are more or less specific to a given species: HAdV types belonging to species B, C, and E predominantly cause respiratory and urinary tract infections, most types in species D cause ocular infections, while species F HAdVs cause gastrointestinal infections. While emerging HAdV types have been responsible for outbreaks that have resulted in hospitalization and fatalities, in healthy adults the course of these illnesses is generally mild and self-resolving [4,5]. The most severe symptoms are associated with types B7 and E4, which cause acute respiratory disease in military recruits, and with several types in species D (mainly D8, 37, and 64) and E4 causing epidemic keratoconjunctivitis. Also, HAdV-F41 was suspected in the etiology of a recent rise in pediatric hepatitis cases [6,7]. HAdVs are very immunogenic; infections result in lifelong immunity against the infecting type. Consequently, the most severe HAdV-related disease occurs in patients with a less functional immune system. HAdV is identified as the causative infectious agent in about one in five newborns and infants hospitalized with pneumonia [8]. Severely immunosuppressed transplant patients are also at risk of developing serious HAdV infections [9]. Pediatric allogeneic hematopoietic stem cell transplant recipients are the most vulnerable: among these patients, disseminated disease can result in an 80% case fatality rate [3,10]. Because of this high vulnerability, special care is taken to shield these patients from pathogens. Still, community-acquired infections and infected donor tissue cannot be ruled out as sources of infection in this patient population [11]. Furthermore, adenoviruses can form persistent, asymptomatic infection in healthy humans that can reactivate upon immunosuppression [11,12]. Disseminated infections are most frequently caused by species C HAdVs, but other HAdV species, irrespective of their tropism in immunocompetent humans, have also been isolated from transplant recipients [3,13,14]. 

Presently, there is no treatment available that has been specifically approved for the treatment of HAdV infection. Besides nonspecific treatments such as human immunoglobulin and temporarily decreasing the intensity of immunosuppression, the most frequently used antiviral drug is cidofovir (CDV), which is approved for the treatment of CMV retinitis [15]. CDV is an acyclic nucleotide phosphonate analogue of cytidine monophosphate [16]. After phosphorylation by cellular kinases, CDV acts both as a chain-terminator of the viral genome and as an inhibitor of the viral polymerase enzyme [17]. Due to its long plasma half-life and because it is a substrate for the human renal organic anion transporter [18], CDV has dose-limiting nephrotoxicity [19]. Several derivatives of CDV have been produced with the aim of reducing its toxicity and increasing its bioavailability. Among these compounds, brincidofovir (BCV), a lipid-linked prodrug reached the farthest point in the development pipeline. In preclinical studies, BCV demonstrated excellent anti-HAdV activity in vitro and in vivo, had improved pharmacokinetics parameters, and significantly reduced kidney toxicity [20,21,22]. However, after promising results in Phase I and Phase II clinical trials, Phase III trials for the prevention of cytomegalovirus (CMV) and HAdV infection had to be halted because patients experienced severe enterotoxicity, which was first misdiagnosed as graft-versus-host disease [23,24,25]. Due to this Phase III result, the development of BCV as an oral drug was abandoned [26]. In 2021, however, BCV was approved by the U.S. Food and Drug Administration with an indication for the treatment of human smallpox disease in adult and pediatric patients, including neonates [27]. 

It is clear that the development of an effective antiviral to treat disseminated HAdV infection is a priority. It is also evident that an animal model is necessary to study the natural history of the infection and to test the efficacy of test compounds. As HAdVs do not replicate in mice or rats, these species cannot be used. Thus, to model HAdV infection and pathogenesis in vivo, we developed the immunosuppressed Syrian hamster model [28]. Syrian hamsters are permissive for the replication of species C HAdV, and the infection causes symptoms that are similar to those seen in human patients [29,30,31,32,33,34]. We and others have been using this animal model to test the efficacy of antiviral compounds [22,35,36,37,38,39,40,41,42,43,44,45,46,47]. 

Here, we show that HAdV-infected Syrian hamsters can be used to model treatment protocols in which the dosing of the antiviral compound is guided by the virus burden. We used two different compounds, NPP-669 and valganciclovir (VGCV), with known efficacy against HAdV infections in the hamster model [35,41,42,47]. To monitor the virus replication longitudinally in the animals, we used a replication-competent HAdV vector in which the luciferase expression is dependent on the viral DNA replication. 

## 2. Materials and Methods

### 2.1. Cells and Viruses

**Scheme 1:**
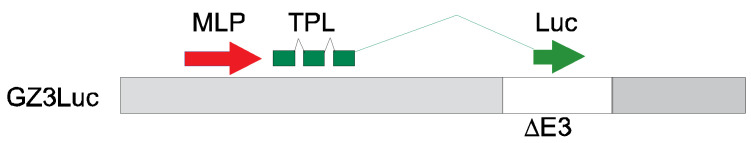
Structure of GZ3Luc. The vector is replication-competent and has a wild-type genome except for the deletion of the E3 region and ectopic expression of the ADP gene (not shown in the figure). The expression of the firefly luciferase gene is driven by the native adenoviral major late promoter (MLP). All the other functions of the MLP (i.e., regulating the expression of late genes) are retained. TPL: tripartite leader; Luc: luciferase gene.

GZ3Luc, also named 007-Luc, a fully replication-competent HAdV that causes pathology in Syrian hamsters similarly to HAdV-C5, was described earlier [48,49]. Briefly, in GZ3Luc, all E3 genes except the Adenovirus Death Protein (ADP) are deleted, and the firefly luciferase gene is inserted such that it is only expressed during the late phase of virus replication, i.e., after viral genome replication (Scheme 1). Thus, the luciferase expression can be used as a surrogate measurement of the virus replication. As most anti-adenoviral compounds presently in development inhibit viral genome replication, this model can be used to test the efficacy of such compounds. As the products of deleted E3 genes function as immunomodulators, their absence is not thought to be detrimental in immunosuppressed hosts [50]. Retaining ADP is expected to contribute to maintaining wild-type levels of pathogenesis by helping with virus spread [51,52]. 

### 2.2. Compounds

NPP-669 (Lot# 16909-CT-32-35) was obtained from TSRL, Inc. (AnnArbor, MI, USA) in powdered form. The drug was suspended in 0.5% carboxymethyl cellulose (Sigma [St. Louis, MO, USA] C5678, Lot# SLBS7273), at a concentration of 0.1 mg/mL to achieve a dose of 1 mg/kg in a 1 mL dose volume for the approximately 100 g hamsters. The drug was made up every three days, aliquoted into daily portions, and stored at 4 °C. Valganciclovir (VGCV; batch 20120410) was purchased from 2A Pharmachem (Lisle, IL, USA), made up in a single batch by dissolving it in water at 20 mg/mL for a dose of 200 mg/kg. The aliquots were stored at 4 °C. For both compounds, the aliquots were allowed to equilibrate to room temperature before dosing. 

### 2.3. Animals

Male Syrian hamsters were purchased from Envigo at 60 to 80 g body weight. For the experiments with immunosuppressed hamsters (depicted in Figures 2–5), the animals were treated with cyclophosphamide (CP). Starting 10 days before challenge, CP was administered intraperitoneally, once at a dose of 140 mg/kg and then twice weekly at a dose of 100 mg/kg for the duration of the experiment. 

The animals were distributed into the required number of groups, with each group containing 9 hamsters. The hamsters in the challenge groups were infected intravenously (i.v.) or intranasally (i.n.) with GZ3Luc at 5 × 10^10^ or 1 × 10^10^ plaque forming units (PFUs)/kg, respectively. The hamsters in some groups were treated for various time intervals, specified for each experiment, with NPP-669 or VGCV. Both drugs were administered orally (p.o.); NPP-669 at 1 mg/kg once daily and VGCV 200 mg/kg twice daily. The body weights and any signs of morbidity in the animals were recorded daily. For certain experiments, serum and liver samples were collected at sacrifice. The virus burden in the liver was determined by 50% tissue culture infectious dose (TCID_50_) assay, and the serum was analyzed for the transaminase levels. Hamsters that became moribund before the termination of the experiment were sacrificed as needed. Besides the animals judged moribund by observation, we sacrificed all the hamsters that lost more than 20% of their original body weight. 

### 2.4. Measuring Radiance

Radiance was measured as described before [48,49]. Briefly, the animals were anesthetized using isoflurane and transferred to the IVIS Spectrum optical-imaging equipment, in which gas anesthesia was maintained. Luminescent images were taken using autoexposure settings. For quantifying the radiance, all the images in an experiment were loaded as a group, and the same size region of interest was drawn, covering the liver, lung, or the nose, respectively, depending on the route of challenge. Radiance was recorded as photons per second (p/s). 

### 2.5. Statistical Analysis 

Statistical analysis was performed using GraphPad Prism 9 (GraphPad Software). Mixed-effects analysis and Šídák’s multiple comparisons test were used to compare the body weight changes and changes in radiance. For the serum transaminase levels and virus burden in the liver, the variance of the samples in all the groups was calculated using the Kruskal–Wallis test, and a comparison between the groups was performed using the two-tailed Mann–Whitney U-test. A *p* ≤ 0.05 was considered significant. 

## 3. Results

### 3.1. GZ3Luc, a Replication-Competent HAdV Vector, Can Be Used to Track Virus Replication In Vivo after Intravenous or Intranasal Challenge 

**Figure 1:**
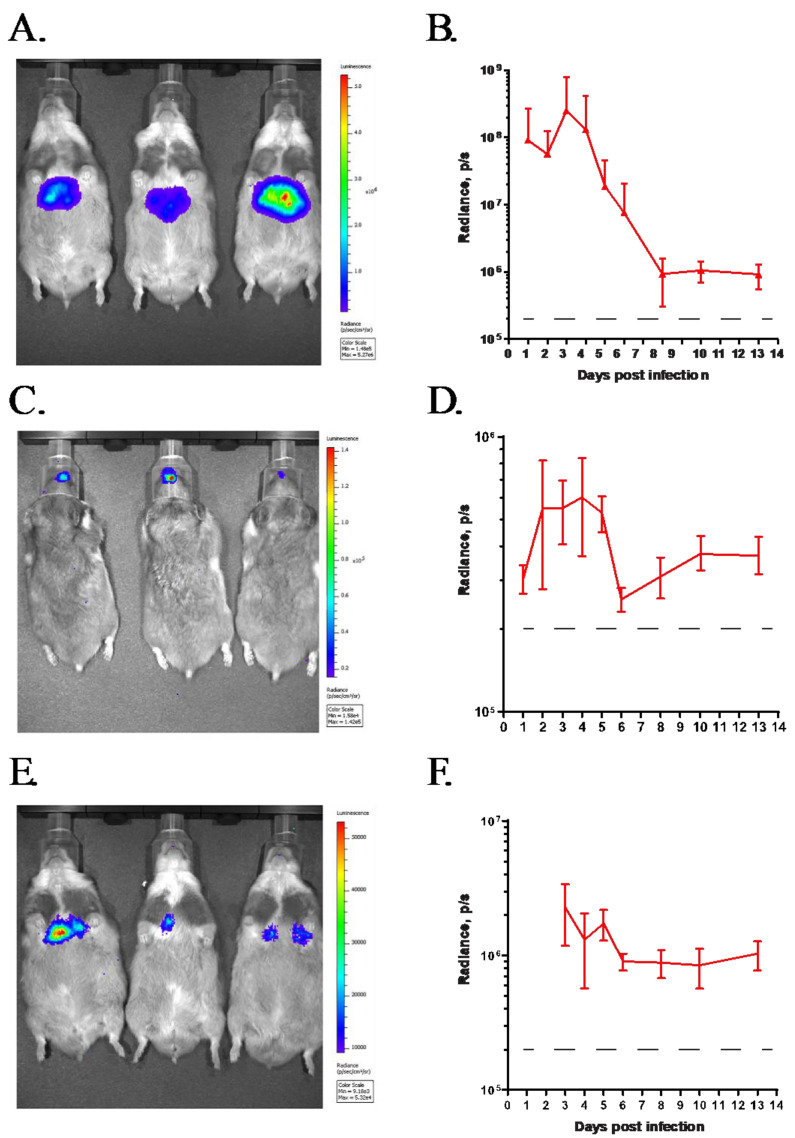
GZ3Luc replicates in the liver, nasal cavity, and lungs of immunocompetent Syrian hamsters. (**A**,**B**) Intravenous challenge. (**C**,**D**) Intranasal challenge, dorsal view. (**E**,**F**) Intranasal challenge, ventral view. The same animals are shown in (**C**,**E**). The dashed line represents the background radiance.

After i.v. administration of GZ3Luc into immunocompetent Syrian hamsters, strong radiance was detected in the mid-abdominal area of the hamsters (Figure 1A). This area coincides with the anatomic location of the liver, which is the main target organ for HAdV replication after i.v. challenge. After an initial increase, the radiance reached peak intensity at 3 days post-challenge, after which it decreased, reaching background levels at around 8 to 10 days post-challenge (Figure 1B). The kinetics of the radiance closely followed the course of the HAdV infection in the hamsters [29]. After i.n. administration of GZ3Luc, we detected radiance in the areas of the rostrum and the thorax, corresponding to the locations of the nasal mucosa and the lung, which are known targets of infection via this route (Figure 1C,D) [29]. The intensity of the radiance in the lung after i.n. inoculation was lower than that in the liver after i.v. infection (Figure 1E). This agrees with our previous observations showing a lower level of HAdV replication in the lungs of hamsters [29,33,40].

### 3.2. HAdV-Induced Pathology Can Be Mitigated with Short-Course Treatment with an Antiviral Compound; However, Low-Grade Virus Replication Persists 

**Figure 2:**
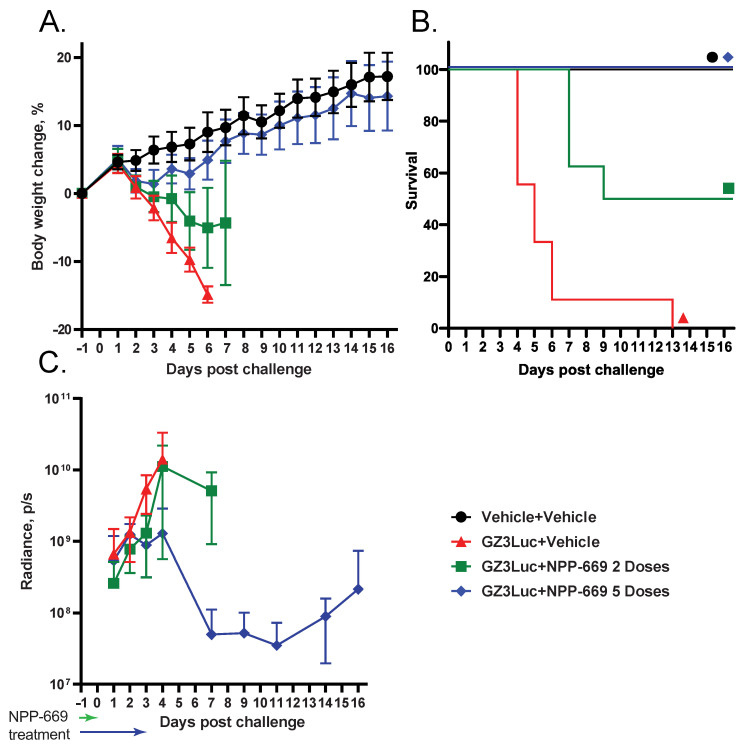
In immunosuppressed hamsters, five doses of NPP-669 mitigate HAdV-induced pathology, and even two doses increase the median survival. Short-course NPP-669 treatment reduces the replication of GZ3Luc; however, virus replication rebounds after 11 days post-challenge. (**A**) Body weight changes. (**B**) Survival. (**C**) Radiance. Radiance (photons/second) was quantified using Living Image 4.7.3 for each animal at each time point. For (**B**,**C**), the group mean and standard deviation are shown. For this and subsequent figures, no group means for the body weight or radiance were calculated after an animal was lost from a given group. The arrows under (**C**) indicate the duration of the NPP-669 treatment.

We reported previously that NPP-669 effectively inhibits HAdV replication and prevents pathology in hamsters when administered daily for the duration of the experiment at 1 mg/kg. To determine the shortest course of treatment with NPP-669 that was still efficacious, we injected immunosuppressed hamsters i.v. with 5 × 10^10^ PFU/kg of GZ3Luc or vehicle (PBS). One GZ3Luc-infected group was treated with drug vehicle, while two other virus-infected groups received daily oral doses of 1 mg/kg of NPP-669, starting one day before virus infection. For one of these groups, treatment was discontinued after two doses (on Day 0), while the other group received five doses (until Day 3). All the GZ3Luc-infected, untreated hamsters were losing body weight starting from day 2, and all the hamsters in this group were sacrificed as moribund (Figure 2A,B). Daily treatment with NPP-669 until 3 days post-challenge mitigated the body weight loss and prevented death (Figure 2B). Two doses of NPP-669 were partially efficacious: the weight loss in this group was not as sudden as in the untreated group, and the median survival was increased from 5 days in the GZ3Luc+Vehicle group to 13 days (*p* < 0.001) (Figure 2A,B). Until 2 days post-challenge, the luciferase expression was similar in all the groups. After this time, the expression increased rapidly in the GZ3Luc-infected, untreated hamsters, while it plateaued and then slowly decreased in the group that received five doses of NPP-669 (Figure 2C). Notably, the luciferase expression increased again after 11 days post-challenge in this group. For the group in which treatment was stopped after day 0, the luciferase expression increased after an initial delay, and at 4 days post-challenge, it was similar to that measured in the untreated group (Figure 2C). 

### 3.3. Persistent HAdV Infection and the Resulting Pathology Can Be Managed with Intermittent Treatment with Antiviral Compounds 

**Figure 3:**
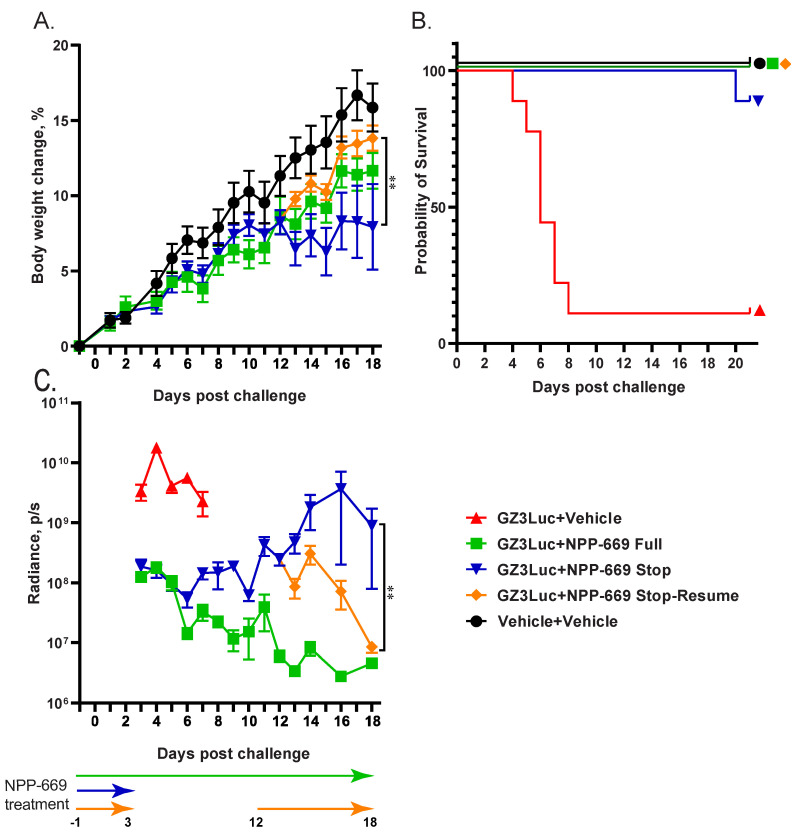
Intermittent treatment of immunosuppressed hamsters with NPP-669 inhibits HAdV replication and mitigates pathology. (**A**) Body weight changes. (**B**) Survival. (**C**) Luciferase expression. The symbols mark the group mean, and the error bars indicate the standard error. The arrows under (**C**) indicate the duration of the NPP-669 treatment for each group. **: *p* < 0.01 (mixed effects analysis).

**Figure 4:**
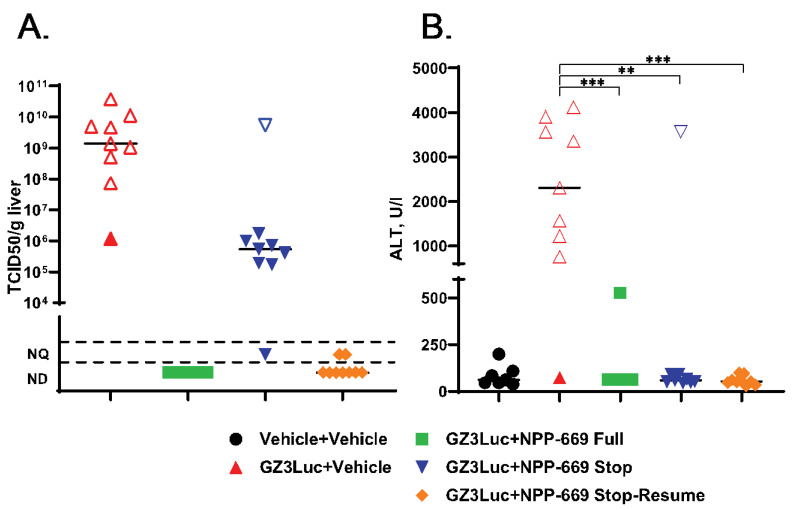
Orally administered NPP-669 suppressed the replication of GZ3Luc in the liver of immunosuppressed hamsters and mitigated liver pathology induced by the infection. (**A**) Virus burden in the liver. (**B**) Serum alanine transaminase (ALT) levels. The symbols indicate data from individual hamsters; empty symbols indicate that an animal was sacrificed ahead of schedule. NQ: not quantifiable; ND: not detectable; **: *p* < 0.01; ***: *p* < 0.001 (Mann–Whitney U-test).

In the previous experiment, we demonstrated that withdrawing NPP-669 treatment 3 days after challenge resulted in the rebounding of virus replication. While the cessation of treatment did not result in significant pathology in the time frame of the experiment, it is to be expected that increasing virus replication will result in organ damage. To investigate this possibility and to assess whether resuming the NPP-669 treatment after increasing the HAdV replication was detected will mitigate pathology, we injected immunosuppressed hamsters i.v. with 5 × 10^10^ PFU/kg of GZ3Luc or vehicle. One of the GZ3Luc-infected groups was treated with drug vehicle, while two other virus-infected groups received daily oral doses of 1 mg/kg of NPP-669, starting 1 day before virus infection. For one such group (*n* = 9), the daily NPP-669 treatments were continued until the conclusion of the experiment. For the other group (*n* = 18), the treatments ceased at 3 days post-challenge (i.e., after five doses). As expected, GZ3Luc replicated unobstructed in the untreated virus-infected hamsters, and these animals lost weight precipitously and all but one succumbed to the infection due to virus replication (Figure 3). Continuous administration of NPP-669 inhibited virus replication and prevented pathology (Figure 3). For the group that received NPP-669 only until 3 days post-challenge, the radiance was slowly rising after an initial decrease, indicating ongoing virus replication. At 12 days post-challenge, this group was split into two, each containing nine randomly selected animals. NPP-669 treatment was restarted in one of these groups, while the other was left untreated. Virus replication increased unabated in the group in which treatment was not resumed, and the hamsters in this group started losing weight, with one hamster eventually dying due to the infection (Figure 3). In contrast, virus replication rapidly decreased in the group for which the NPP-669 treatment was restarted (Figure 3C), and these animals gained weight similarly to their counterparts in the uninfected group (Figure 3A). The hamsters were sacrificed and necropsied at the culmination of the experiment or when they became moribund. The moribund hamsters showed marked pathological lesions characteristic of adenovirus infection, while no significant gross-necropsy findings were noted for any of the hamsters sacrificed at the conclusion of the experiment (day 21). Corroborating the radiance data, a high virus burden was found in the liver of the untreated hamsters (Figure 4A). Continuous or intermittent treatment inhibited GZ3Luc replication, while an intermediate level of virus burden was detected in the liver of hamsters that received NPP-669 up to 3 days post-challenge (Figure 4A). There was a strong correlation between the virus burden and the radiance values (r = 0.8204, *p* < 0.0001; Spearman nonparametric correlation). The serum samples were analyzed for the serum alanine aminotransferase (ALT) level as a surrogate for liver damage. Untreated GZ3Luc infection caused an elevation of the ALT levels, which was significantly mitigated by NPP-669 (Figure 4B). No statistically significant difference was seen between the groups for which the treatment was continued or interrupted.

**Figure 5:**
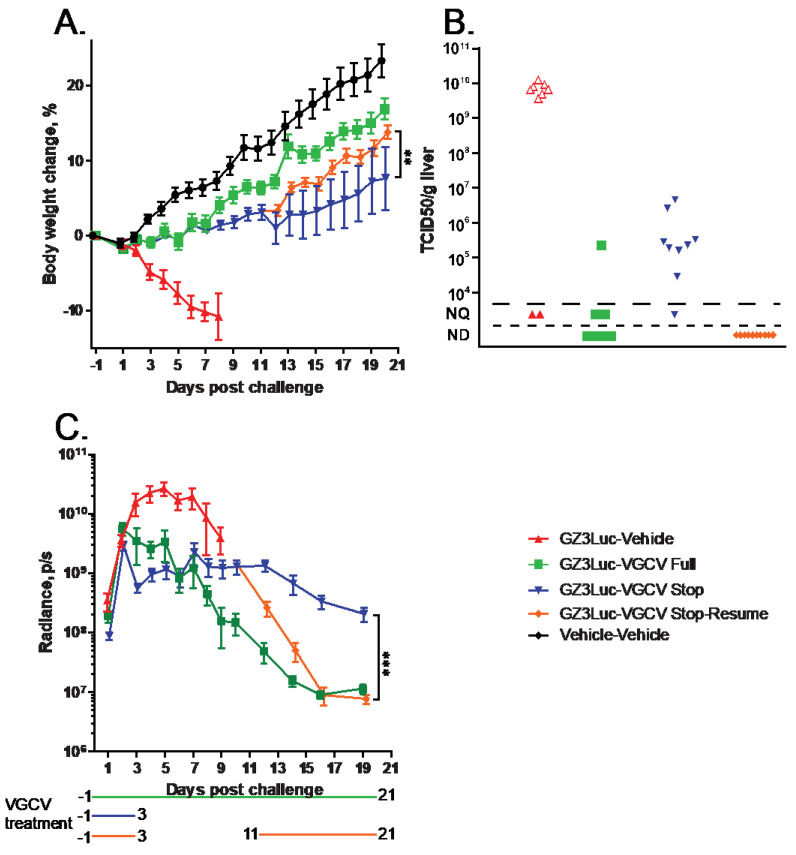
Intermittent treatment with VGCV inhibits HAdV replication and mitigates pathology. (**A**) Body weight changes. (**B**) Infectious virus burden in the liver at sacrifice. The symbols represent data from individual hamsters; empty symbols indicate that an animal was sacrificed ahead of schedule. NQ: not quantifiable; ND: not detectable; **: *p* < 0.01, ***: *p* < 0.001 (mixed effects analysis). (**C**) Luciferase expression. For (**A**,**C**), the symbols mark the group mean, and the error bars indicate the standard error. The lines under (**C**) indicate the duration of the NPP-669 treatment for each group.

We obtained similar results when we used VGCV to treat GZ3Luc-infected immunosuppressed hamsters. For the untreated hamsters, radiance peaked at 5 days post-challenge, after which it declined (Figure 5C). These hamsters lost weight rapidly (Figure 5A) and eventually succumbed to the infection. Continuous VGCV treatment suppressed virus replication, and these hamsters were gaining weight similarly to the uninfected ones. In the group in which VGCV treatment was halted at 3 days after challenge, after an initial decrease, GZ3Luc replication plateaued or even slightly increased between days 4 and 11 (Figure 5C). For half of the hamsters in this latter group, twice daily VGCV treatment was resumed at 11 days post-challenge. For these hamsters, the luciferase expression decreased quickly, while the decline was slower for the hamsters that did not receive VGCV (Figure 5C). Notably, resuming drug administration at 11 days post-challenge significantly increased the body weight gain compared to the counterparts that received VGCV for only 5 days (Figure 5A). Analysis of the liver of untreated hamsters that were sacrificed moribund at 8 days post-challenge revealed a very high virus burden, in concert with the high radiance detected in these hamsters (Figure 5B). Continuous administration of VGCV suppressed virus replication to undetectable/unquantifiable levels, while an intermediate virus burden was seen in the liver of hamsters that received VGCV for 5 days (Figure 5B). Notably, resumption of drug treatment at 11 days post-challenge eliminated GZ3Luc from the liver (Figure 5B).

## 4. Discussion

In clinical practice, the strategy of performing virologic surveillance in transplant recipients followed by pre-emptive antiviral therapy for patients who reach certain viral burden thresholds aims to mitigate end-organ disease while also reducing the overall exposure to antiviral agents [53]. Within these high-risk populations, a higher viral load is associated with end-organ disease as well as increased morbidity and mortality [54,55]. Administration of pre-emptive antiviral therapies in response to increasing viral loads detected during routine virologic monitoring has been shown to be effective in preventing symptomatic disease [56]. Due to the limitations of available antiviral therapies, pre-emptive strategies are not well established for HAdV infections, but studies suggest that early detection of an increasing HAdV viral load and subsequent initiation of an antiviral agent, along with the reduction of immunosuppression, is effective in preventing HAdV disease [57,58]. At this point, we do not have sufficient data to determine the influence of various treatment regimens on the emergence of resistant virus strains. Clearly, more research is needed to address this question.

To provide a model to evaluate such treatment schedules, we turned to the immunosuppressed hamster model. To monitor the course of infection in these animals, we needed a method to assess the change in the virus burden in the animals and to correlate it to the pathology (i.e., mortality and body weight loss). As repeated sample collection from the same animal is not feasible in this model, we employed a replication-competent HAdV vector named GZ3Luc, in which the luciferase expression is dependent on the virus replication. As the half-life of luciferase is ~6 h and it requires ATP to function, changes in the radiance measured in the animals during the study can be used as a surrogate for ongoing virus replication. A similarly engineered herpesvirus vector was successfully used to follow the replication of varicella-zoster virus in humanized mice [59]. Initial experiments with GZ3Luc showed that after both i.v. and i.n. infection, the localization of radiant areas in the animals agreed with the tropism of wild-type adenovirus. The natural history of GZ3Luc infection is similar to that of wild-type HAdV-C5: in immunocompetent hamsters, virus replication (based on the radiance values) peaked at around 3 days post-challenge, after which it rapidly declined, causing no excessive pathology. In contrast, virus replication increased unchecked in immunosuppressed hamsters, eventually resulting in the death of the majority of the animals. Treatment with antiviral agents suppressed the replication of GZ3Luc and mitigated the pathology. The radiance levels could be correlated to the body weight loss throughout the experiment and to the infectious virus burden at the conclusion of the study. Based on these data, we argue that the immunosuppressed Syrian hamster infected with luciferase-expressing replication-competent HAdV vector system is usable for longitudinal monitoring of in vivo HAdV infection and pathogenesis and to assess the efficacy of interrupted antiviral treatment regimens.

To model such a treatment regimen, we chose two antiviral compounds, NPP-669 and VGCV. NPP-669, a prodrug of CDV, is highly efficacious against disseminated HAdV infections in the hamster model [35,47]. Merely two doses, the first 1 day before and the second on the day of challenge, were enough to significantly improve the median survival, while five doses of the drug completely prevented mortality and weight loss. VGCV is an orally available prodrug of ganciclovir that has a lower, but still significant, efficacy than NPP-669 against HAdV in the hamster model [41,42]. The mechanism of action of both compounds is to inhibit viral genome replication, which can be monitored by luciferase expression/radiance in GZ3Luc-infected hamsters. As reported previously, the continuous administration of either compound effectively suppressed virus replication and prevented pathology. In hamsters for which we stopped treatment after 5 days of dosing, virus replication continued at a low rate without apparent pathogenesis for a short period. However, approximately a week after the cessation of treatment, virus replication increased and the weight gain of the animals slowed down compared to the uninfected or continuously treated ones. Resuming drug administration at this time was sufficient to suppress virus replication and mitigate pathology. Restarting treatment completely eliminated the infectious virus, while almost all the surviving hamsters in the group that received treatment for only 5 days had infectious virus in their liver. With the exception of one hamster, these latter animals had a virus burden in the medium range, and their body weight gain was significantly slower than that of the controls; however, their serum transaminase levels were in the normal range. These data suggest a low-grade, persistent HAdV infection that was sufficient to cause only minimal pathology. There was one outlier in this group; in this hamster, virus replication increased rapidly after 11 days post-challenge, and the hamster eventually succumbed to the infection. At sacrifice, it had a very high virus burden and a high serum alanine aminotransferase level. Why this animal had such enhanced virus replication and pathology is unclear. However, it demonstrates that there is an ongoing, active virus replication in these hamsters that can flare up in certain individuals.

The course of systemic HAdV infection in hamsters is faster than that in humans: while in transplant patients the infection can persist for months, hamsters either become moribund at around 5 to 7 days post-challenge, or the virus replication burns itself out in about two weeks and the hamster recovers [11,29,60]. There is also a noticeable threshold effect for the dose required to cause pathology: a very high challenge dose is necessary and the difference between a no-effect and lethal dose is just several fold. This is attributed to the presence of tissue-resident macrophages, primarily Kupffer cells, that take up most of the injected bolus of virus but are not permissive for virus replication [29,61,62]. Thus, this is a dead-end infection, and the macrophage compartment needs to be saturated before the infection overflows to the permissive hepatocytes. We speculate that a difference between the immunosuppressive regimens of human patients and the one we employ for hamsters may cause the difference in the progression of the disease. Hamsters are immunosuppressed with a short course of high-dose cyclophosphamide treatment. This is not enough to cause a decrease in the number of tissue macrophages with lifespans of months. Conversely, transplant patients receive long-term immunosuppressive treatment that can result in the depletion of macrophages, thus destroying an important barrier to the infection. Despite these differences, we argue that this animal model can be used to model the reactivation of a low-grade persistent infection and to assess the efficacy of various treatment regimens. 

## Data Availability

All data generated is shown in the manuscript.
